# Efficacy and Outcome Predictors of Gonadotropin Treatment for Male Congenital Hypogonadotropic Hypogonadism: A Retrospective Study of 223 Patients

**DOI:** 10.1097/MD.0000000000002867

**Published:** 2016-03-07

**Authors:** Zhaoxiang Liu, Jangfeng Mao, Xueyan Wu, Hongli Xu, Xi Wang, Bingkun Huang, Junjie Zheng, Min Nie, Hongbing Zhang

**Affiliations:** From the Department of Endocrinology, Key Laboratory of Endocrinology, National Health and Family Planning Commission, Peking Union Medical College Hospital (ZL, JM, XW, HX, XW, BH, JZ, MN), and Department of Physiology, State Key Laboratory of Medical Molecular Biology, Institute of Basic Medical Sciences, Peking Union Medical College and Chinese Academy of Medical Sciences (HZ), Beijing, China.

## Abstract

Gonadotropin induces masculinization and spermatogenesis in men with congenital hypogonadotropic hypogonadism (CHH). However, large cohort studies for the efficacy and reliable predictors of this therapy need to be conducted. The aim of this study was to investigate the efficacy of gonadotropin treatment in a large cohort of male CHH patients and analyze putative predictors for successful spermatogenesis.

This retrospective study included 223 CHH azoospermic patients without puberty development treated between 2005 and 2014. All patients received combined human chorionic gonadotropin (HCG) and human menopausal gonadotropin (HMG) and were followed up for >6 months (5109 person-months). Serum total testosterone level, testicular size, spermatogenesis, and pregnancy outcome were recorded at each visit.

After gonadotropin therapy, testicular size was enlarged from 2.1 ± 1.6 to 8.1 ± 4.6 mL (*P* < 0.001) and serum total testosterone was elevated from 0.9 ± 0.5 to 15.1 ± 8.2 nmol/L (*P* < 0.001). Spermatogenesis (>0/mL) occurred at a median period of 15 months (95% confidence interval 13.5–16.5). Larger basal testicular volume (*P* = 0.012) and noncryptorchidism history (*P* = 0.028) are independent predictors for earlier sperm appearance. Sixty four percent (143/223) of patients succeeded in producing sperms and the average time for initial sperm detection was 14 ± 8 months. However, their sperm concentrations (11.7 [2.1, 24.4] million/mL) and sperm progressive motility (A + B 36.9% ± 20.2%) are significantly lower than World Health Organization standards. Of the 34 patients who desired for fathering children, 19 patients impregnanted their partners during the treatment.

Gonadotropin therapy induces spermatogenesis in male CHH patients. A larger basal testicular size and noncryptorchidism history are favorable indicators for earlier spermatogenesis.

## Abstract 2

Supplemental Digital Content is available in the text

## INTRODUCTION

Congenital hypogonadotropic hypogonadism (CHH), caused by deficiency or dysfunction of gonadotropin-releasing hormone (GnRH), is a disorder characterized by delayed puberty development and infertility. The incidence of CHH is 1–10:100,000 in live births. CHH is comprised of Kallmann syndrome and normosmic CHH (nCHH). Kallmann syndrome is a subgroup of CHH associated with a defective sense of smell (anosmia or hyposmia).^1^ When fertility is required, pulsatile GnRH infusion or combined human chorionic gonadotropin (HCG) and human menopausal gonadotropin (HMG) therapy may promote spermatogenesis.^2^ Combination of gonadotropins can also induce spermatogenesis in patients with acquired hypogonadotropic hypogonadism (HH) of various causes, such as surgery for pituitary tumors, sellar radiation, and sellar craniopharyngioma.^3,4^

Most studies regarding gonadotropin-induced spermatogenesis did not stratify patients into CHH and acquired HH and therefore the effective rate for CHH alone is largely unknown.^5–8^ A recent meta-analysis showed that the overall rate of gonadotropin-induced spermatogenesis in HH was 75%.^9^ However, the success rate of achieving spermatogenesis is significantly lower in prepubertal-onset HH patients, as compared with a mixed population of pre- and postpubertal onset patients (68% [58–77] vs 84% [76–89], *P* = 0.011).^9^ Warne et al^8^ reported a study achieving near 80% success rate of spermatogenesis in an unspecified HH group with the largest sample size (n = 77). The rate of gonadotropin-induced spermatogenesis in CHH patients could be lower than that of acquired and CHH combined. For example, a study of 74 CHH patients treated with gonadotropins showed a success rate of 65%.^10^ Currently, data on spermatogenesis of CHH patients is still very limited due to the low incidence of this disease. The success rate and its influencing factors of gonadotropin-induced spermatogenesis need to be further demonstrated in large cohort studies for CHH patients.

In the present work, we retrospectively reported the fertility and paternity outcomes in 223 CHH patients treated with gonadotropins (HCG/HMG) and analyzed putative predictors for successful spermatogenesis.

## METHODS

### Patients

CHH azoospermic patients without puberty development were included into this retrospective study. Diagnosis of CHH was made if a patient met all of the following criteria: a male aged over 18 years without puberty development, a serum testosterone level <100 ng/dL (3.5 nmol/L) with low or normal levels of gonadotropins, normal levels of other pituitary hormones and negative findings in sellar magnetic resonance imaging (MRI).^11^ Clinical presentations, cryptorchidism, medical history as well as family history were recorded. Tests of plasma gonadotropins and testosterone, MRI of pituitary and the olfactory nerve were performed. The study protocol was reviewed and approved by the ethics committee of Peking Union Medical College Hospital.

**FIGURE 1:**
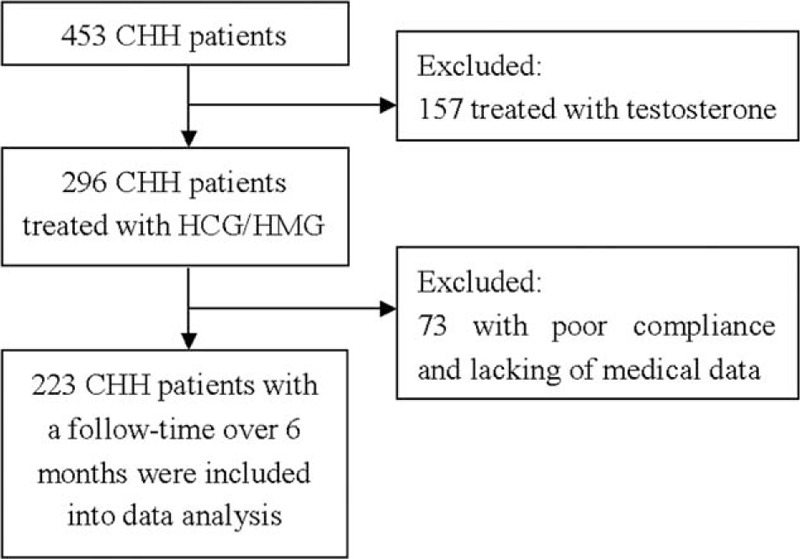
Flowchart of screening patients. CHH = congenital hypogonadotropic hypogonadism.

From January, 2005 to December, 2014, a total of 296 male CHH patients were treated with HCG/HMG in Peking Union Medical College Hospital. There were 73 cases excluded from data analysis for poor compliance or incomplete medical data. Thus, only 223 patients were retrospectively evaluated (Figure 1).

### Treatment and Follow-up

Patients discontinued androgen therapy (if used) for at least 3 months before starting gonadotropin therapy. Intramuscular HCG (2000–5000 U, Livzon Pharmaceutical Co, Guangdong, China) injection twice weekly was given for 6 months. Then HMG (75–150 U, Livzon Pharmaceutical Co.) was intramuscularly injected twice weekly. Regular follow-ups were conducted at an interval of 3 to 6 months. The dosages of gonadotropins were adjusted according to plasma testosterone level and sperm output. Testicular size, plasma gonadotropins, plasma testosterone, and sperm count were measured on each visit.

The levels of testosterone were determined 48 hours after HCG injection. Luteinizing hormone (LH), follicle-stimulating hormone (FSH), and testosterone levels were measured by chemiluminescent method using a commercial kit (ACS 180 Automatic Chemiluminescence System; Bayer, Germany). Testicular size was measured using a Prader orchidometer and the mean value of bilateral testicular volumes was used in data analysis. Semen samples were collected by masturbation and analyzed according to the standard World Health Organization (WHO) method (before 2010, according to 1999 WHO method, after 2010, according to 2010 WHO method).^12^ Sperm motility was classified as fast progressive sperm (A), slow progressive sperm (B), nonprogressive sperm (C), and immotile sperm (D). The proportion in each of the 4 motility categories was assessed.

### Outcomes

Successful spermatogenesis, as a primary outcome, was defined as the first sperm detected by microscopy. The secondary outcomes were specific sperm concentrations and conception. Four sperm thresholds, >0 (any sperm appeared under microscopy), >5, >10, and >15 million/mL, were recorded according to the sperm concentration. Self-reported pregnancy was noted.

### Statistical Analysis

SPSS version 17.0 was used for data analysis (SPSS Inc, Chicago, IL). Normal distributive data were expressed as the mean ± SD, and non-normal distribution data were listed as median (quartiles). The paired *t* test was used to compare the difference of the plasma testosterone and testicular volume before and after the treatment. Kaplan-Meier analyses were used to estimate the median time for achieving different sperm thresholds. Cox regression models were built to analyze the predictors of successful spermatogenesis. A multivariate linear regression model was built to identify the contributing factors on the time for patients achieving spermatogenesis. The independent samples *t* test was used to compare the baseline difference between Kallmann and nCHH patients, as well as patients with spermatogenesis (S subgroup) and without spermatogenesis (nonspermatogenesis subgroup, N subgroup). The different rate of cryptorchidism and family history between different groups was compared by *χ*^*2*^ test. Statistical significance was set at *P* < 0.05.

Age for initiating HCG/HMG treatment, body mass index (BMI), peak LH after GnRHa (triptorelin, 100 μg) stimulation, family histories of delayed puberty (0 = no, 1 = yes), histories of cryptorchidism (0 = no, 1 = unilateral, 2 = bilateral), basal testicular volume, previous androgen exposure (0 = none or <3 months, 1 = >3 months), and previous gonadotropins exposure (0 = none or <3 months, 1 = >3 months) were considered as variables in Cox regression model and multivariate linear regression model.

## RESULTS

### Clinical Characteristics of CHH Patients

A total of 223 male CHH patients were included for analysis. The mean follow-up period is 23 ± 13 months (5109 person-months totally). Gonadotropin treatment was initiated at the age of 22.4 ± 2.3 years. Baseline serum levels of LH, FSH, and testosterone were 0.3 ± 0.5 IU/L, 0.9 ± 0.9 IU/L, and 0.9 ± 0.5 nmol/L, respectively. Mean testicular volume was 2.1 ± 1.6 mL. Forty patients (18%) had a history of cryptorchidism (18 unilateral and 22 bilateral) and 18 (8%) had a family history of delayed puberty (Table 1). They were generally in good conditions with normal blood and urine routine test results, and normal liver and renal function. Thyroid hormones, adrenal glucocorticoids, and growth hormones levels were all in the normal range. During the gonadotropin therapy, their plasma testosterone increased from 0.9 ± 0.5 to 15.1 ± 8.2 nmol/L (*P* < 0.001) and testicular volume enlarged from 2.1 ± 1.6 to 8.1 ± 4.6 mL (*P* < 0.001) (by paired *t* test).

**TABLE 1:**
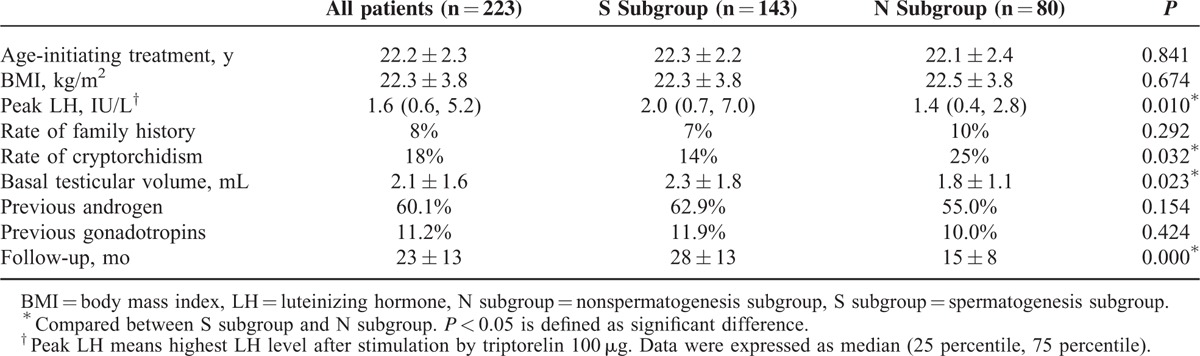
Baseline Features of Congenital Hypogonadotropic Hypogonadism Patients

Of all the patients, half (111/223) were having Kallmann syndrome, whereas another half (112/223) were having nCHH. Comparison between the 2 groups (by independent samples *t* test and *χ*^*2*^ test) showed that patients with Kallmann syndrome were prone to have a family history (17/111 vs 1/112, *P* < 0.001). However, Kallmann syndrome and nCHH patients had similar BMI, age of initiating treatment, peak LH level after triptorelin stimulation, basal testicular volume, and rate of cryptorchidism. At the last visit, testicular volume of Kallmann patients was 6.9 ± 4.2 mL (follow-up period 21 ± 12 months), whereas testicular volume of nCHH patients was 9.3 ± 4.6 mL (follow-up period 25 ± 14 months) (see Table 1, Supplemental Content, which illustrates the comparison between the 2 groups).

### Spermatogenesis Outcomes in CHH Patients

**FIGURE 2:**
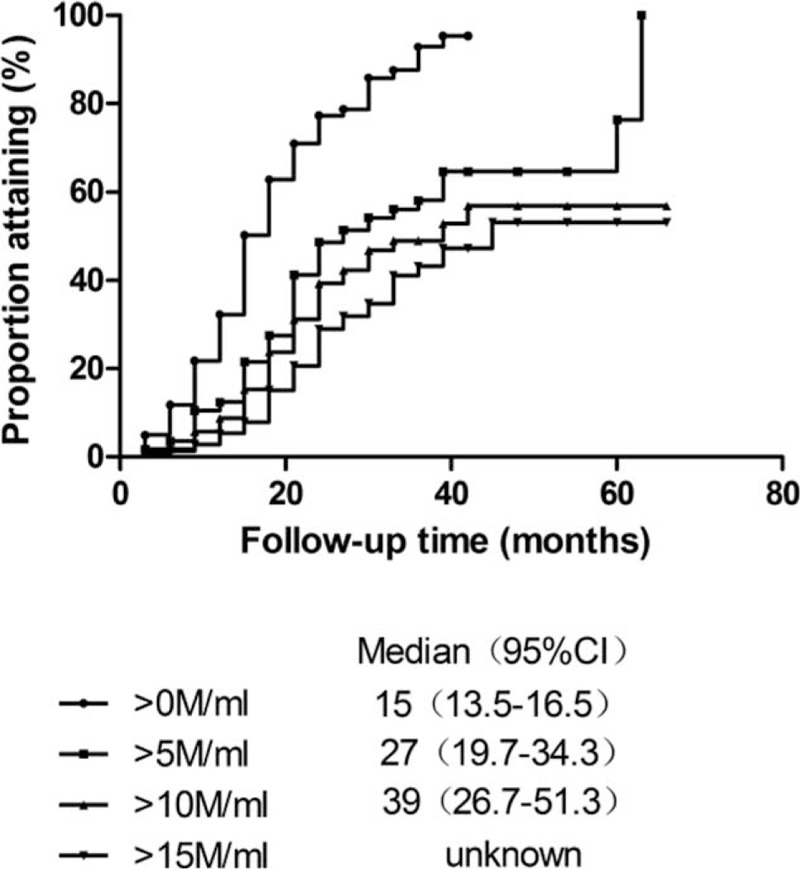
Median times of achieving sperm concentration at different thresholds. Kaplan-Meier analysis of 223 patients showed median times of achieving sperm concentration more than 0, 5, 10, 15 million/mL. The median time of first spermatogenesis was 15 months. The median and 95% CIs for time to reach 15 million/mL cannot be estimated. CI = confidence interval, M/mL = million/mL.

Kaplan-Meier analysis showed that median time for the first sperm appearance of all the patients (n = 223) was 15 months (95% CI, 13.5–16.5), with an average testicular volume of 7.1 ± 3.0 mL. To reach sperm threshold >5 and >10 million/mL, the median time were 27 (95% CI 19.7–34.3) and 39 months (95% CI 26.7–51.3), with an average testicular volume of 9.5 ± 3.2 and 9.9 ± 3.2 mL, respectively. The estimated median time for sperm concentration >15 million/mL could not be obtained because of limited number of patients who produced sperms above this level (Figure 2). The median time of initial successful spermatogenesis was similar between patients with Kallmann syndrome and nCHH (18 vs 15 months, *P* = 0.136, Log Rank [Mantel-Cox]). According to Cox-related method (including age initiating treatment, BMI, peak LH, family history, cryptorchidism, basal testicular volume, prior androgen, prior gonadotropins), larger basal testicular volume (β = 0.142, *P* = 0.012) and noncryptorchidism history (β = 0.421, *P* = 0.028) were 2 favorable predictors for a shorter time to achieve spermatogenesis (Table 2).

**TABLE 2:**
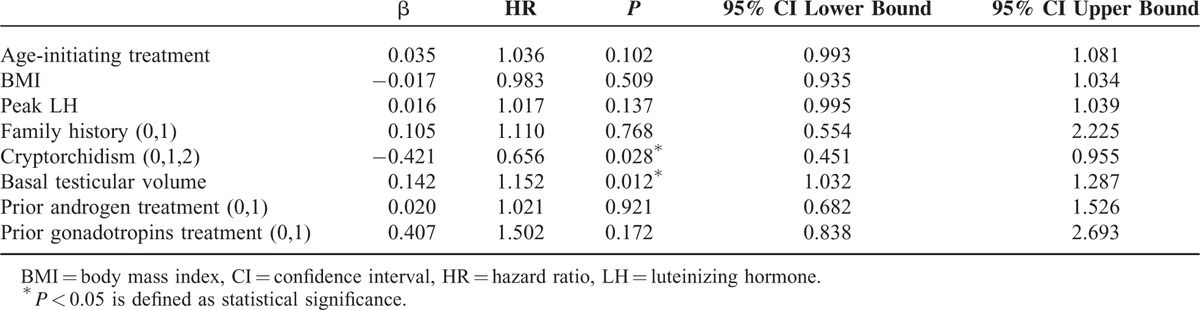
Predictors for Spermatogenesis (Correlated Cox Analysis)

**FIGURE 3:**
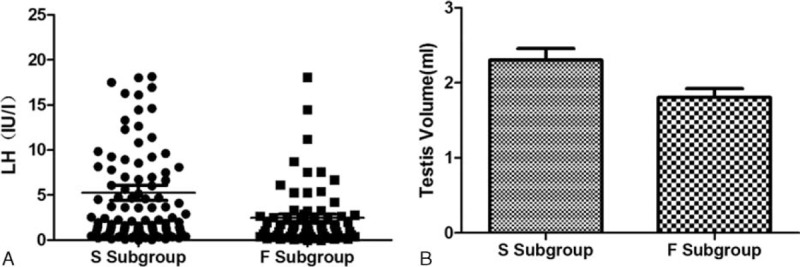
Peak LH and basal testicular volume in patients with or without spermatogenesis. Comparison of peak LH (stimulated by triptorelin 100 μg) and basal testicular volume between patients with spermatogenesis and without spermatogenesis. LH = luteinizing hormone, N subgroup = nonspermatogenesis subgroup, S subgroup = spermatogenesis subgroup.

For the 143 patients (64%, 143/223) who succeeded in spermatogenesis, the mean time of first sperm detection is 14 ± 8 months. Twenty-nine patients found sperms for the first time after 6 months’ treatment, 42 patients after 12 months, 44 patients after 18 months, 15 patients after 24 months, 8 patients after 30 months, 4 patients after 4 months and 1 patient did not get sperms until 42 months. Compared with those who failed sperm production (N subgroup), the successful group (S subgroup) had a lower rate of cryptorchidism (25% vs 14%, *P* = 0.032, by *χ*^*2*^ test), larger basal testicular volume (1.8 ± 1.1 vs 2.3 ± 1.8 mL, *P* = 0.023, by independent *t* test) and higher peak LH level after triptorelin stimulation (Figure 3, Table 1). Patients in S group had a longer follow-up period than that in N group (28 ± 13 vs 15 ± 8 months, *P* < 0.001, by independent *t* test). Multivariate linear regression model (including age for initiating HCG/HMG treatment, BMI, peak LH after GnRHa stimulation, family histories of delayed puberty, histories of cryptorchidism, basal testicular volume, previous androgen exposure and previous gonadotropins exposure) showed that age of initiating treatment (β = −0.445, *P* = 0.002), basal testicular volume (β = −1.074, *P* = 0.003) and cryptorchidism histories (β = 3.457, *P* = 0.004) were major predictors for earlier spermatogenesis following the treatment (Table 3), indicating that older patients with larger basal testicular size and noncryptorchidism history may have less time to produce sperms.

**TABLE 3:**
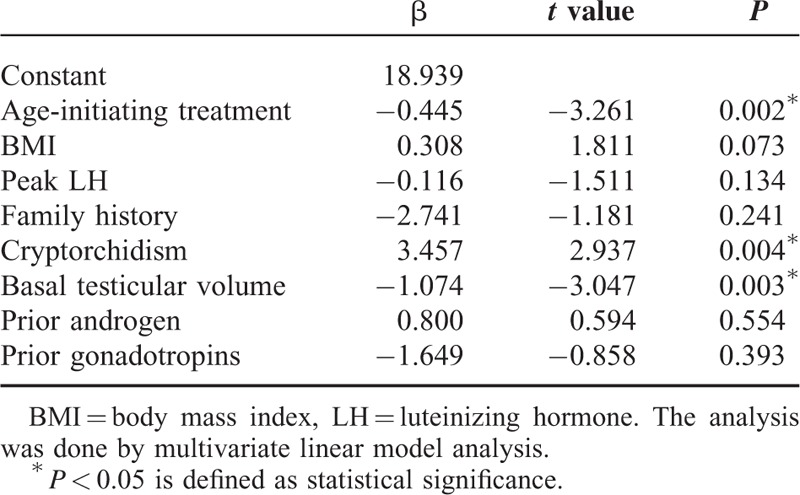
Influential Factors on Time of Achieving the First Sperm in Spermatogenesis Subgroup (n = 143)

Patients in S subgroup had a sperm concentration of 11.7 (2.1, 24.4) million/mL at the end of follow-up and was lower than that of healthy Chinese young men (50.0 [36.0, 63.8] million/mL) (*P* < 0.001).^13^ The sperm progressive motility (A + B) ([36.9% ± 20.2%] vs [52.6% ± 11.2%], *P* < 0.001) and total mobility (A + B + C) ([44.4% ± 21.9%] vs [58.6% ± 11.1%], *P* < 0.001) were significantly lower than that of healthy young men (n = 1808, age ranges 22–30 years).^13^ According to WHO reference values for human semen,^14^ 36% (51/143) of our patients attained normal sperm concentrations (≥15 million/mL). Among them, only 67% (34/51) had normal sperm-progressive motility (A + B) (≥32%).

In S subgroup, 34 patients were married and 20 pregnancies occurred in 19 (19/34) wives, including 19 natural conceptions (one woman had two pregnancies) and 1 in vitro fertilization (IVF). Seven natural conceptions happened with sperm concentrations of 1 million/mL, 6 happened between 1 and 10 million/mL, 3 happened between 10 and 20 million/mL, 2 happened with sperm concentration >20 million/mL (see Table 2, Supplemental Content, which presents sperm concentrations of CHH patients at the time that natural conceptions happened). Fourteen infants, including 7 girls and 7 boys, were delivered with normal appearance of external genitalia. Six abortions happened, including 4 miscarries (karyotype 45, XO was confirmed in one fetus), and 2 induced abortions because of parents’ concern of transmitting the disease to their offspring.

### Safety Evaluation

During the period of study, gynecomastia was developed in 7% (16/223) of the subjects and 2 of them had mammary excision. Acne occurred in 9% (20/223) of the patients and was subsequently alleviated after reducing HCG dosages. No hepatorenal impairment or allergic reactions were reported.

## DISCUSSION

To analyze the efficacy of gonadotropin treatment and identify putative predictors for successful spermatogenesis in a large cohort of male CHH patients, we conducted a retrospective study of 223 patients treated at our hospital. For all the patients, 64% of them succeeded in spermatogenesis. Basal testicular size and cryptorchidism history are predictors of patients’ response to the treatment.

The median time for achieving the first sperm after the initial treatment in our cohort is 15 months, which is much longer than previously reported 6 to 12 months.^5,8,15–17^ The discrepancy may be attributable to the following factors. First, subjects recruited in most of previous studies comprised not only CHH but also acquired HH patients. Acquired HH patients are easier to achieve spermatogenesis because of their larger testicular size and higher Leydig and Sertoli cells reservoir.^18^ Second, to improve patients’ compliance, HMG was injected at 75 to 150 IU and twice weekly in our study, compared with 75 to 225 IU and 3 times weekly in other studies. The lower dosage and less frequency may take longer time for spermatogenesis. Third, the HMG used in our study was extracted from the urine of postmenopausal women and may not have an efficacy comparable with recombinant FSH proteins used in other studies.^19^

Although spermatogenesis or even pregnancy is achievable in some of CHH patients after gonadotropins treatment, sperm concentration and mobility are usually far below normal levels.^8,9,17^ Similarly, the sperm concentration and mobility induced by gonadotropins in our study are lower compared either with WHO standards ^14^ or with that reported in healthy Chinese men.^13^ This phenomenon may be largely explained by absence of mini puberty, which leads to a decreased reservoir for Sertoli and Leydig cells in CHH patients.^18^ Furthermore, mutations in certain genes, such as *FGFR1*^20,21^ and *KAL1,*^22,23^ have a direct impairment on testicular development and spermatogenesis. Last but not least, unlike endogenous pulsatile and fine-tuning production of gonadotropin in healthy men, optimal spermatogenesis might not be achieved by intermittent injection of an un-physiological high level of HCG/HMG in CHH patients.

Studies have shown that basal testicular volume and cryptorchidism history are key indicators for successful spermatogenesis.^3,5,6,8,24^ Our data also suggest that larger basal testicular size confers earlier spermatogenesis, whereas cryptorchidism requires a longer time for attainment of sperms. Patients with bilateral cryptorchidism had a lower rate of spermatogenesis than that of unilateral cryptorchidism.

Warne et al^8^ reported that patients with a low BMI (<30 kg/m^2^) had a greater chance of achieving spermatogenesis compared with men with a BMI > 30 kg/m^2^. Liu et al^5^ suggested that a history of androgen exposure was associated with poorer therapeutic response, which was not observed in other studies.^9^ Previous gonadotropin use was associated with an earlier sperm appearance in Liu et al's study,^5,25^ which was not supported by Warne et al’ study.^8^ BMI and history of androgen or gonadotropins exposure did not influence spermatogenesis outcome in the present study. Prospective studies are thus needed to determine the potential role of these factors in spermatogenesis.

In our study, a larger basal testicular size, a lower incidence rate of cryptorchidism, and a higher GnRHa-stimulated LH level were observed in the patients with spermatogenesis. It is known that these parameters may reflect a better function of hypothalamic-pituitary-gonadal axis in CHH patients.^26^ A multiple linear analysis in our spermatogenesis subgroup further confirmed that a larger basal testicular size and noncryptorchidism were favorable factors for earlier spermatogenesis. Interestingly, an older age for starting HCG/HMG treatment was associated with an earlier sperm appearance. One possible explanation is that older patients were more eager for fertility and thus had better compliance of the treatment. Comparing with the nonspermatogenesis group, longer follow-up of the patients in spermatogenesis group may be attributed to satisfactory outcome of spermatogenesis and/or better compliance of the treatment. Moreover, some patients need long time to get a sperm (even 42 months); thus, it should be known that persistent treatment is necessary sometimes.

Of 34 married patients who desired for children, 56% of them impregnated their partners during the therapy. However, these pregnancies were associated with 20% natural abortion, which is higher than that in normal population (8%–17%).^27,28^ The higher rate of natural abortions may be because of the presence of aberrant sperms from the patients.^29^ Two induced abortions were carried out because of the couples’ concern about potential heredity of CHH. Genetic information on CHH patients and fetus are warranted for family planning.^2,4^ Fourteen babies with normal genital phenotype were born in our study. Their hypothalamus pituitary gonadal axis function needs to be examined for an early detection of potential CHH inherited from their parents.

No severe adverse events were observed during gonadotropin therapy in our study. Acne and gynecomastia occurred in <10% of the patients. The former was caused by increased serum testosterone and alleviated by reducing HCG dosage. The latter was induced by estrogens aromatized from increased testosterone. Surgical removal of the obviously enlarged breast tissue may be an option for these patients.^17^ Overall, gonadotropin therapy is safe for the patients with CHH.

In this study, we achieved spermatogenesis in 64% of 223 CHH patients with gonadotropin treatment. Larger basal testicular volume and a noncryptorchidism history are major favorable indicators for earlier sperm appearance. With the largest sample size of CHH reported in the literature, our study provides strong evidence for the efficacy and responsive factors of gonadotropin in induction of spermatogenesis for CHH patients. Several issues may be addressed in future studies. Pathogenic genetic mutations in CHH patients should be screened, as certain mutations may be the major determinants of spermatogenesis. The genetic information may also be used in prenatal diagnosis. Mini puberty in neonates should be monitored as it may provide information about hypothalamus pituitary gonadal function of the patients’ offsprings.

## Supplementary Material

Supplemental Digital Content
