# Supplementary material for: Efficacy and Outcome Predictors of Gonadotropin Treatment for Male Congenital Hypogonadotropic Hypogonadism: A Retrospective Study of 223 Patients
Source: Medicine (Baltimore). 2016 Mar 7;95(9):e2867. doi: 10.1097/MD.0000000000002867 (PMC4782854; doi:10.1097/MD.0000000000002867)
Supplement: Supplemental Digital Content [file medi-95-e2867-s001.pdf]

Table 1. Comparison between patients with Kallmann syndrome and normosmic congenital hypogonadotropic hypogonadism (nCHH).

	**Kallmann (n=111)**	**nCHH (n=112)**	***P* value**
**Age initiating treatment (y)**	22.1 ± 5.3	22.4 ± 5.3	0.674
**BMI (Kg/m^2)**	21.9±3.6	22.8±3.9	0.084
**Peak LH (IU/L) #**	1.4(0.6, 4.5)	2.0 (0.6, 7.5)	0.227
**Rate of family history**	15% (17/111)	0.01% (1/112)	< 0.001*
**Rate of cryptorchidism**	23% (25/111)	13% (15/112)	0.054
**Basal testicular volume (ml)**	1.9±1.3	2.3±1.8	0.078
**Ultimate testicular volume (ml)**	6.9 ± 4.2	9.3 ± 4.6	< 0.001*
**Follow-up (months)**	21 ± 12	25 ± 14	0.017*
**Median time for initial spermatogenesis (months)**	18	15	0.136^

nCHH = normosmic congenital hypogonadotropic hypogonadism, BMI = body mass index, LH = luteinizing hormone

*Compared between patients with Kallmann syndrome and nCHH. *P* < 0.05 is defined as significant difference. # Peak LH means highest LH level after stimulation by triptorelin 100μg. Data was expressed as median (25 percentile, 75 percentile)

^ Log Rank (Mantel-Cox).

Table 2. Sperm concentrations of CHH patients at the time that natural conceptions were succeeded (17 paitents with 18 conceptions).

Patient number	Sperm concentration (million / ml)
**1**	9.39
**2**	6.79
**3**	1.0
**4**	12.26
**5**	17.56
**6**	6.96
**7**	1.0
**8**	33.12
**9**	1.0
**10**	1.0
**11**	21.0
**12**	1.0
**13**	7.28
**14**	1.0
**15**	18.16
**16**	1.0
**17**	3.25
**18**	9.21

CHH = congenital hypogonadotropic hypogonadism; Number 10 and 11 refer to the same patient.
